# Impact of SIV infection on mycobacterial lipid-reactive T cell responses in Bacillus Calmette-Guérin (BCG) inoculated macaques

**DOI:** 10.3389/fimmu.2022.1085786

**Published:** 2023-01-16

**Authors:** Edith M. Walker, Kristen M. Merino, Nadia Slisarenko, Brooke F. Grasperge, Smriti Mehra, Chad J. Roy, Deepak Kaushal, Namita Rout

**Affiliations:** ^1^ Division of Microbiology at Tulane National Primate Research Center, Covington, LA, United States; ^2^ Southwest National Primate Research Center, Texas Biomedical Research Institute, San Antonio, TX, United States; ^3^ Department of Microbiology and Immunology, Tulane University School of Medicine, New Orleans, LA, United States; ^4^ Tulane Center for Aging, Tulane University School of Medicine, New Orleans, LA, United States

**Keywords:** BCG, SIV, lipid antigen, γδT, tuberculosis, macaque

## Abstract

**Background:**

Although BCG vaccine protects infants from tuberculosis (TB), it has limited efficacy in adults against pulmonary TB. Further, HIV coinfection significantly increases the risk of developing active TB. In the lack of defined correlates of protection in TB disease, it is essential to explore immune responses beyond conventional CD4 T cells to gain a better understanding of the mechanisms of TB immunity.

**Methods:**

Here, we evaluated unconventional lipid-reactive T cell responses in cynomolgus macaques following aerosol BCG inoculation and examined the impact of subsequent SIV infection on these responses. Immune responses to cellular lipids of *M. bovis* and *M. tuberculosis* were examined ex vivo in peripheral blood and bronchioalveolar lavage (BAL).

**Results:**

Prior to BCG inoculation, innate-like IFN-γ responses to mycobacterial lipids were observed in T cells. Aerosol BCG exposure induced an early increase in frequencies of BAL γδT cells, a dominant subset of lipid-reactive T cells, along with enhanced IL-7R and CXCR3 expression. Further, BCG exposure stimulated greater IFN-γ responses to mycobacterial lipids in peripheral blood and BAL, suggesting the induction of systemic and local Th1-type response in lipid-reactive T cells. Subsequent SIV infection resulted in a significant loss of IL-7R expression on blood and BAL γδT cells. Additionally, IFN-γ responses of mycobacterial lipid-reactive T cells in BAL fluid were significantly lower in SIV-infected macaques, while perforin production was maintained through chronic SIV infection.

**Conclusions:**

Overall, these data suggest that despite SIV-induced decline in IL-7R expression and IFN-γ production by mycobacterial lipid-reactive T cells, their cytolytic potential is maintained. A deeper understanding of anti-mycobacterial lipid-reactive T cell functions may inform novel approaches to enhance TB control in individuals with or without HIV infection.

## Introduction

Tuberculosis (TB) disease caused by *Mycobacterium tuberculosis* (Mtb) remains a major cause of infectious disease mortality worldwide. In 2020, there were about 10 million new cases and 1.3 million deaths, which is the first annual increase in the number of people dying from TB since 2005 (1). Moreover, HIV infection remains a significant risk for developing TB, accounting for up to 21% of all TB-related deaths in persons with HIV (2). *Mycobacterium bovis* Bacille Calmette-Guérin (BCG) is the only licensed vaccine that protects children from disseminated TB but has shown limited efficacy against pulmonary TB in adults (3, 4). The outcome of Mtb infection ranges from latent infection to overt TB disease depending on differences in the immune response (5). However, the protective immune mechanisms in individuals that clear or control Mtb infection remain unknown, thereby impeding effective vaccine development. Thus, deciphering the early events in anti-mycobacterial immune responses in a prospective manner, particularly in lungs as the primary site of natural infection, is crucial for prevention of TB.

T cells play a key role in controlling Mtb infection (6–8). Multiple studies have provided important insights into the adaptive immune responses of CD4 T cells in TB immunity (9–11). Distinct T lymphocytes with different cytokine profiles contribute to either resistance or susceptibility to TB (12). For instance, Type 1 responses by CD4 T cells, including the induction of IFN-γ, IL-2, and cytolytic activity, have been shown to be important in mycobacterial immunity (13–16). Further, IFN-γ responses have been associated with protective mycobacterial immunity in active and latent TB (17–19), as well as in PBMC from BCG-vaccinated individuals (17–20). Besides, Th1-type CD4 T cell responses, early γδ Tcell, invariant natural killer T cell (iNKT), natural killer (NK) cell, B-cell, neutrophil, myeloid dendritic cell (mDC), and mucosal-associated invariant T (MAIT) cell responses are also observed following BCG inoculation (12). Additionally, mounting evidence points toward the anti-mycobacterial potential of unconventional CD1-restricted T cell responses targeting Mtb lipid antigens *via* Th1-type and cytotoxic effector functions (21–24). Mtb lipid vaccination in guinea pigs demonstrated induction of CD1-restricted responses and reduced lung pathology after Mtb challenge (25, 26), suggesting a role of these responses in TB immunity. Yet, owing to challenges of studying their functions in early immune responses in humans, and due to limited experimental tools and differences from humans in CD1 gene isoforms in guinea pigs besides the absence of group 1 CD1 genes in mice, it is not clear how lipid antigen-specific T cells mediate these protective effects *in vivo*. Macaques exhibit the full spectrum of clinical TB (active, latent, and reactivation TB) and display substantial homology with the human CD1 system (27), making them a good model to investigate the *in vivo* role of lipid-reactive T cell responses in mycobacterial immunity.

BCG has been studied as an experimental model for mycobacterial infection and antimycobacterial immunity in earlier studies (28–30). In this study, we utilized macaques exposed to aerosol BCG as a model to understand the dynamics of mycobacterial lipid-reactive T cell responses in peripheral blood and the airways. Frequencies, phenotypic and functional characteristics of unconventional T cell populations were characterized in peripheral blood and BAL fluid before and after BCG inoculation. We further evaluated the impact of SIV infection on lipid-reactive T cell functions in peripheral blood and airways of BCG-inoculated macaques. Our findings demonstrate the presence of innate-like mycobacterial lipid antigen-specific T cells in nonhuman primates that display distinct kinetics of response in the airways to live mycobacterial exposure than that of conventional peptide antigen-specific T cell responses and may contribute to systemic anti-mycobacterial cytolytic functions during chronic SIV infection.

## Methods

### Ethics statement

Animals in this study were housed at the Tulane National Primate Research Center (TNPRC), accredited by the Association for Assessment and Accreditation of Laboratory Animal Care (AAALAC) International. The study was approved by the TNPRC Institutional Animal Care and Use Committee (IACUC; P0359-2017) and was conducted under the standards of the US National Institutes of Health Guide for the Care and Use of Laboratory Animals. The NIH Office of Laboratory Animal Welfare assurance number for the TNPRC is A4499-01. Following BCG inoculation, animals were housed in Animal Biosafety Level 2 indoor housing. All animal procedures including BCG and virus administration, sample collection, and euthanasia were carried out under the direction of TNPRC veterinarians.

### Animals and infection

Six healthy male Mauritian cynomolgus macaques ranging in age from 3 to 9 years old and seronegative for SIV, HIV-2, STLV-1 (Simian T Leukemia Virus type-1), SRV-1 (type D retrovirus), and herpes-B viruses were used in this study. The macaques were socially housed in pairs after enrollment in the project. Animals were inoculated with Bacillus Calmette–Gueírin (BCG)-Danish at 1000 CFU by aerosol inoculation as earlier described (31). Briefly, a custom head-only dynamic inhalation system housed within a class III biological safety cabinet was used for this purpose (32), which is an established model of aerosol Mtb inoculation in macaques (33). Three animals were administered with 10^6^–10^7^ TCID50 SIVmac251 challenge stocks (obtained from the Preclinical Research and Development Branch of Vaccine and Prevention Research Program, Division of AIDS, NIAID) *via* intravenous (iv) injection. Three animals were similarly injected with saline and treated as the sham-injected SIV-negative control group.

### Bacterial burden and viral load quantification

The presence of live BCG was evaluated in bronchoalveolar lavage (BAL) collected at 3-, 5-, and 7-weeks post aerosol BCG inoculation, and at 3-, 5-weeks post-SIV inoculation and at necropsy as earlier described (31). Briefly, BAL fluid was used as neat, or 10-times diluted in sterile PBS and plated in triplicate for incubation at 37°C. CFU were counted manually after 4 weeks of incubation to calculate BCG CFU/mL. Blood samples in EDTA vacutainer tubes (Sarstedt Inc Newton, NC) were taken for a complete blood count and routine chemical analysis and centrifuged within one hour of phlebotomy for plasma separation. SIV Plasma viral load quantification was performed using Roche High Pure Viral RNA Kit (Catalog #11858882001) as earlier described (34). The limit of detection of SIV RNA was 83 copies per milliliter of plasma.

### Blood and BAL processing

Blood PBMCs were isolated using Lymphocyte Separation Medium (MP Biomedicals Inc., Solon, OH) with standard procedures. Briefly, blood was centrifuged at 14000*g* for 5 min at 4°C and plasma aliquots were cryopreserved at − 80°C until used. Subsequently, PBMC were separated by density gradient centrifugation at 1825*g* for 20 min at 20°C using deceleration without braking and used for phenotyping and *in vitro* functional assays. BAL wash fluid (2 × 20 ml washes of sterile PBS) obtained from macaque lungs was centrifuged at 950g for 10 minutes at 4°C to pellet cells and subsequently washed and re-suspended in complete RPMI-10 medium containing 10% FCS before fluorescent antibody staining or stimulation for functional assays.

### Multiparameter flow cytometry

Multi-color flowcytometric analysis was performed on cells according to standard procedures using anti-human mAbs that cross-react with cynomolgus macaques. For phenotype analysis, PBMC were surface stained with CD3 (BD, SP34-2), CD4 (BD, L200), CD8 (BD, SK1), CD14 (BD, M5E2), CD20 (Biolegend, 2H7), HLA-DR (Biolegend, L243), CD127 (Beckman Coulter, R34-34), CD161 (Biolegend, HP-3G10), CD45 (BD, D058-1283), CD183 (Biolegend, G025H7), and TCR γδ (BD, B1), PBS-57-loaded CD1d Tetramers (CD1d TM) (NIH Tetramer core). Surface staining was carried out by standard procedures as earlier described (35). Intracellular cytokine staining (ICS) assays were carried out on mycobacterial lipid antigen-stimulated PBMCs. Following 16 h incubation, cells were washed in PBS containing 2% FCS and 0.5 mM EDTA and stained for surface markers in wash buffer for 30 min at 4°C. The cells were then washed and permeabilized using the BD Cytofix/Cytoperm reagent for 20 min at 4°C and washed with BD Perm/Wash Buffer and underwent staining for intracellular markers. Antibodies used for these assays included surface markers listed above as well as CD69 (Biolegend, FN50), IFN-γ (Biolegend, 4S.B3), and granzyme B (lifetechnologies, GB12). Cells were finally washed in wash buffer and fixed in 1% paraformaldehyde in PBS. Flow cytometric acquisition was performed on the BD Fortessa instrument driven by the FACS DiVa software for at least 200,000 CD3+ T cells in PBMC or at least 50,000 CD3+ T cells for BAL lymphocytes. The data acquired were analyzed using FlowJo software (version 10.8.1; TreeStar, Ashland, OR).

### Functional analyses

Ex vivo analysis of secreted IFN-γ and Perforin was carried out by ELISPOT assay (Mabtech) in lymphocytes from blood and tissues at an input of 2 × 10^5^ cells/well that were directly stimulated with *M. bovis* Strain AF 2122/97 total lipids (TL), Mtb H37Rv TL, Mtb H37Rv lipoarabinomannan (LAM), Mtb H37Rv Phosphoinositol mannoside-6 (PIM-6), Mtb H37Rv mycolic acids (MA), Mtb H37Rv Culture Filtrate Proteins (CFP), and Mtb TB10.4 peptide array obtained from BEI Resources, NIAID, NIH. For γδ T cells and NKT cells, (E)-4-hydroxy-3-methyl-but-2-enyl pyrophosphate (HMBPP; Sigma-Aldrich, Saint Louis, MO) and α-galactosylceramide (α-GalCer; Avanti Polar Lipids, Alabaster, AL) were used as stimulating antigens. The capture- and biotinylated detector-matched mAb pair for IFN-γ were clones MT126L and 7-B6-1, respectively and for Perforin were clones Pf-80/164 and Pf-344, respectively. Briefly, unfractionated lymphocytes were plated on anti–IFN-γ/Perforin–coated sterile 96-well polyvinylidene difluoride MultiScreen-IP plates (Millipore, Bedford, MA) and stimulated overnight with antigens. Spots were developed by successive incubation with streptavidin-alkaline phosphatase followed by the substrate NBT/5-bromo-4-chloro-3-indolylphosphate buffer (Moss, Pasadena, MD). Spots were counted on a KS ELISPOT Automated Reader System (Carl Zeiss, Thornwood, NY) using KS ELISPOT 4.2 software (performed by ZellNet Consulting, Fort Lee, NJ). Frequencies of responding cells obtained after subtracting background spots in negative control wells were expressed as spot-forming cells (SFC) per million PBMC. ELISPOT responses to individual stimulating antigens that were >2-fold above those of negative control wells, and >50 SFC/10^6^ cells were considered positive. Alternatively, 24 h culture supernatants were collected from PBMC stimulated with lipid antigens at 1 × 10^6^ cells/ml in 48-well cell culture plates and stored frozen at −20°C. TNF-α, and IFN–γ secretion was detected in supernatants by using ELISA for monkey cytokines (U-CyTech, Utrecht, Netherlands), performed according to the manufacturer’s instructions. Levels of cytokines (pg/mL) were interpolated from standard curves.

### Statistical analyses

All statistical analysis was performed using GraphPad Prism Software (Version 9.4.1). Data were analyzed by analysis of variance (ANOVA) with multiple comparisons. Tukey’s *post hoc* test was used for multiple group comparisons and Dunnett’s *post hoc* test was used for comparison of different time-points with pre-BCG baseline. Wilcoxon matched pairs signed rank test was used to compute longitudinal differences in phenotypic markers. Unpaired t test was used to compute differences in cytokine and cytolytic granule secretion in SIV-infected or uninfected groups with respect to pre-SIV/sham baseline. Asterisks indicate significant differences between time points. *P* values of 0.05 or lower were considered significant, ^∗^p<0.05, ^∗∗^p<0.01, ^∗∗∗^p<0.001.

## Results

### Lipid-reactive T cell responses to mycobacterial antigens in BCG-naïve macaques

**Figure 1:**
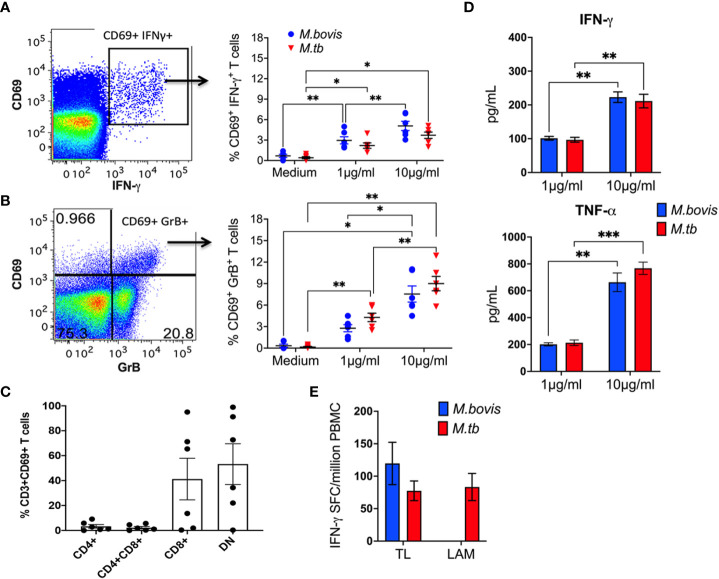
Lipid-reactive T cell functions in peripheral blood of BCG-naïve macaques. Representative flow cytometry plots of CD3+ T lymphocytes in PBMC showing intracellular IFN-γ **(A)** and GrB **(B)** staining on recently activated CD69+ T cells. Frequencies of CD69^+^IFN-γ^+^ and CD69^+^GrB^+^ cells are shown for PBMC isolated from 5 BCG-naïve macaques and stimulated for 12h with 1μg/ml or 10μg/ml TL fractions of *M.bovis* (blue circles) or *Mtb* (red triangles). Medium alone served as negative control. **(C)** CD4 and CD8 co-receptor expression denoted as CD4^+^, CD8^+^, CD4^+^CD8^+^ double positive, and double negative (DN) on Mtb TL-responsive CD69+ T cells was quantified using cryopreserved PBMC obtained from 6 BCG-naïve macaques. **(D)** IFN-γ responses following stimulation with *Mtb* TL and LAM (red bars) and *M.bovis* TL (blue bar) expressed as SFU/million PBMCs. **(E)** IFN-γ and TNF-α ELISA showing total amounts of Th1 cytokines secreted by *Mtb* TL (red bar) and *M.bovis* TL (blue bar) reactive T cells, expressed as pg/ml. Data shows mean and SEM and are representative of two independent experiments. Doses were compared using repeated measures two-way ANOVA with Tukey’s *post hoc* tests. Asterisks indicate significant differences between doses (*p < 0.05; **p < 0.01; ***p < 0.001).

Based on the innate immune functions of NKT cells and γδ T cells, the two well characterized subsets of CD1-restricted T cells, a conceptual model has emerged on the innate-like functionality of unconventional T cells that respond to non-peptide antigens. Thus, we investigated innate-like responses to mycobacterial lipid antigens in TB-naïve macaques. *In vitro* stimulation of PBMC with total cellular lipid fractions isolated from *M. bovis* and *M. tuberculosis*-H37Rv resulted in T cell upregulation of CD69 as an early activation marker (representative plots in Figure A-B). Further, 12h stimulation with mycobacterial lipids induced greater intracellular IFN-γ and GranzymeB (GrB) production by CD69^+^ T cells in a dose-dependent manner (**Figures 1A, B**; right). The CD69^+^ T cells responding to mycobacterial lipids displayed a predominantly CD8^+^ and CD4/CD8 double negative (DN) T cell phenotype and rare usage of CD4 coreceptor (**Figure 1C**), suggesting a potential role in effector functions. IFN-γ ELISPOT revealed the presence of IFN-γ secreting cells in response to the *M. tb*-H37Rv cell wall lipid LAM, and total lipids from *M. bovis* and *M.tb* (**Figure 1D**). Additionally, Th1-type cytokine secretion by lipid-reactive T cells was confirmed in the stimulated cell culture supernatants using IFN-γ and TNF-α ELISA, with a higher concentration of mycobacterial lipids resulting in the release of significantly greater amounts of the cytokines (**Figure 1D**). No significant differences in cytokine production were observed between cells stimulated with *M. bovis* and *M. tb* total lipids. These data indicate the presence of circulating mycobacterial lipid-reactive T cells with Th1-type and cytolytic potential in naïve macaques.

### Early increase in frequencies of BAL γδ T cells following aerosol BCG exposure

**Figure 2:**
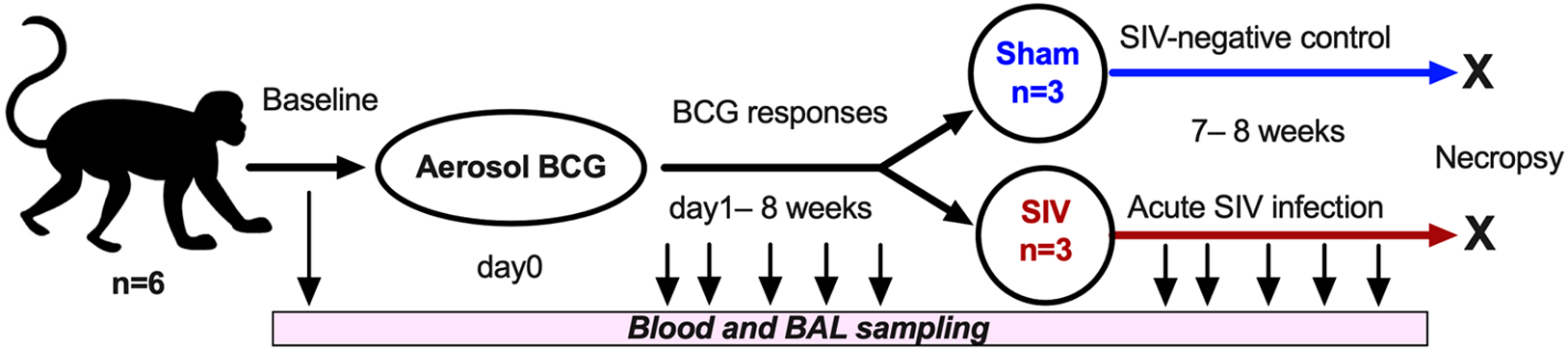
Study Design. Cynomolgus macaques (CM, n=6) were inoculated *via* aerosol exposure with a target dose of 1,000 CFU of *M. bovis* BCG (Danish). Serial collections of blood were obtained at several study timepoints, including d0, d1, and then weekly post-BCG inoculation till necropsy. SIVmac251 was inoculated intravenously into 3 test animals and 3 control animals received saline injection (sham) at 8 weeks-post-BCG, and the animals underwent euthanasia at 7 weeks post-SIV challenge infection.

To understand the influence of *in vivo* mycobacterial exposure on lipid-reactive T cell responses and the impact of subsequent SIV infection on these responses, we next used an aerosol BCG inoculation model followed by intravenous SIV_mac251_ challenge. Macaques were inoculated with BCG-Danish *via* aerosol as earlier described (31) and blood and BAL fluid were collected at serial time-points to evaluate peripheral blood and airway mucosal immune responses (**Figure 2**). Body temperature and weight were recorded through the course of the study at day-1 and weekly post-inoculation till necropsy at 15-16 weeks post-BCG (**Supplementary Figure 1**). Aerosol exposure to BCG did not induce dyspnea, anorexia or significant changes in body temperatures relative to pre-infection values.

**Figure 3:**
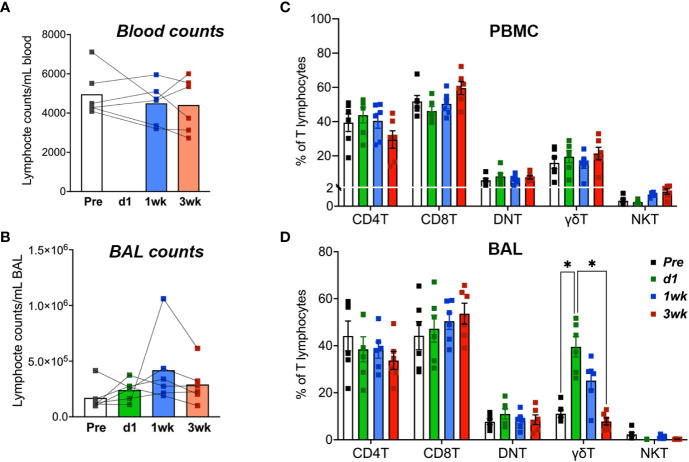
T Lymphocyte numbers in blood and BAL fluid following aerosol BCG exposure. Absolute counts of **(A)** blood lymphocytes and **(B)** BAL lymphocytes at pre-BCG, day1 (d1)-, 1- and 3-week post-BCG inoculation of cynomolgus macaques. Frequencies of T lymphocyte subsets including CD4 T, CD8 T, DNT, γδ T cells, and NKT cells determined by flow cytometry in **(C)** PBMC, and **(D)** BAL cells at the indicated time-points. Data shows mean and SEM. Time-points were compared using repeated measures two-way ANOVA with Tukey’s *post hoc* tests. Asterisks indicate significant differences in frequency of cell subsets between time-points (*p < 0.05).

No significant changes were observed in blood lymphocyte counts following BCG inoculation, although there was a trend of increase in lymphocyte numbers in BAL fluid by 1-week post-inoculation (**Figures 3A, B**). Similarly, in peripheral blood T cells, no significant differences in frequencies of CD4 T, CD8 T, DNT, γδ T cells, and NKT cells were noted following BCG exposure (**Figure 3C**). However, in BAL fluid, the frequencies of γδ T cells rapidly increased at day-1 and returned to baseline by 3 weeks post-inoculation (**Figure 3D**). The presence of live mycobacteria was evaluated in BAL fluid from the BCG-inoculated macaques at 3-, 5- and 8-weeks post-BCG time-points. No BCG was recovered from BAL at any of these time-points post-BCG suggesting clearance of the bacterium in all animals by 3 weeks of aerosol exposure.

### BCG-induced increase in IL-7R and CXCR3 expression by unconventional T cells

**Figure 4:**
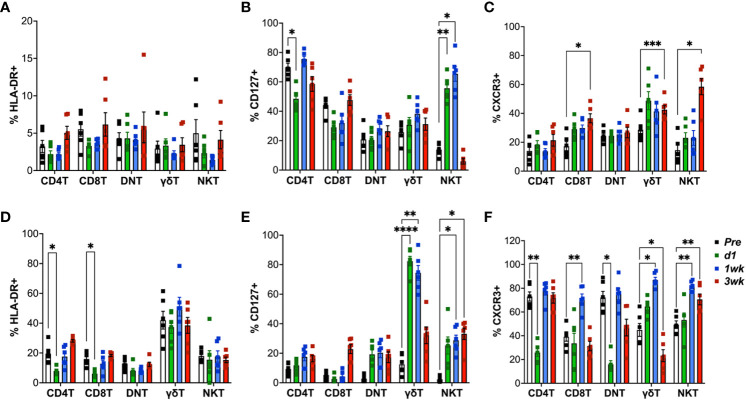
Expression of HLA-DR, CXCR3 and CD127 (IL-7R) on T cell subsets following aerosol BCG exposure. Frequencies of CD4 T, CD8 T, DNT, γδ T, and NKT cells in PBMCs (top panel) and BAL cells (bottom panel) expressing HLA-DR **(A, D)**; CD127 **(B, E)**; CXCR3 **(C, F)** at pre-BCG inoculation (white bars) and d1 (green bars), 1wk (blue bars), and 3wk post-BCG (red bars) time-points. Comparisons of different time points with respect to pre-BCG baseline using repeated measures one-way ANOVA with Dunnett’s *post hoc* tests. Asterisks indicate significant differences between time points (*p < 0.05; **p < 0.01; ***p < 0.001; ****p < 0.0001).

In order to elucidate early T cell responses in blood and airways following aerosol BCG inoculation in macaques, we stained cells for expression of HLA-DR, CXCR3 and CD127. In the first 3 weeks following BCG inoculation, there was no significant increase in expression levels of the activation marker HLA-DR on circulating T cells (**Figure 4A**). On the contrary, there was an early decrease HLA-DR expression of classical CD4T and CD8T cells in BAL fluid that returned to baseline by week-2 (**Figure 4D**). Since Interleukin (IL)-7 signaling is essential to T-cell proliferation and function, and the IL-7 receptor (IL-7R/CD127) expression on CD8 T cells is associated with development of fully protective immune memory (36), we evaluated longitudinal CD127-expression post-BCG inoculation. CD127 expression increased only in unconventional lipid-reactive T cell subsets, which included NKT cells in PBMC, and both γδ T cells and NKT cells in BAL between 1-2 weeks (**Figures 4B–E**). Notably, the increase in CD127^+^ γδ Tcell frequencies was highly significant in BAL at d1 (P<0.001) and 1-week (P=0.001) that returned to baseline by 3-week time-point, when BCG was likely cleared as no live bacteria was recovered from BAL fluid at that time. Further, expression of the Th1 marker, CXCR3, on blood γδ T cells, NKT cells, and CD8T cells showed a trend of early increase that was significant at week-3 post-BCG (**Figure 4C**). A similar early increase in CXCR3 expression was observed on BAL γδ T cells, NKT cells and CD8T cells that was significant at week-2 post-BCG, suggesting induction of early Th1-type responses at the site of BCG exposure (**Figure 4F**). CXCR3 returned to baseline in γδ T cells and CD8T cells but stayed elevated in NKT cells at 3 weeks post-BCG.

### Enhanced IFN-γ response of mycobacterial lipid-reactive T cells in peripheral blood and airways following aerosol BCG inoculation

**Figure 5:**
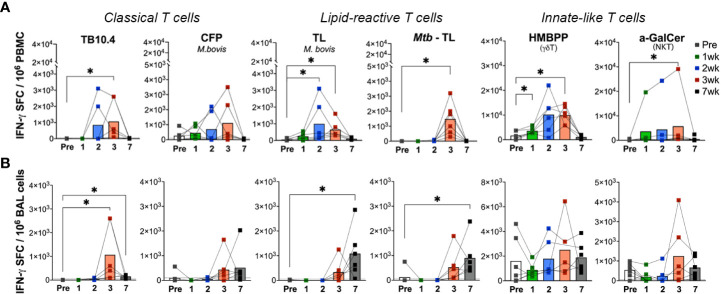
Kinetics of peptide- and lipid-reactive IFN-γ responses in blood and BAL following aerosol BCG exposure. IFN-γ responses expressed as SFU/million cells, following stimulation with *Mtb* TB10.4 peptide pool, *M.bovis* culture filtrate proteins (CFP), *M.bovis* and *Mtb* TL fractions, E-4-hydroxy-3-methyl-but-2-enyl pyrophosphate (HMBPP), and alpha-galactosylceramide (α-GalCer) in freshly isolated **(A)** PBMCs, and **(B)** BAL lymphocytes. Pre-BCG inoculation (white bars) and 1wk (green bars), 2wk (blue bars), 3wk post-BCG (red bars), and 7wk (grey bars) time-points were evaluated. Wilcoxon matched pairs signed rank test was used to compute difference between time points with respect to pre-BCG baseline. Asterisks indicate significant differences between time points (*p < 0.05).

To further examine the effects of aerosol BCG exposure on systemic and local lipid-reactive T cell functions, we evaluated their IFN-γ and perforin producing ability in PBMC and BAL fluid. Ex vivo lipid reactive T cell responses were evaluated by using total lipids (TL) fractions isolated from *M.bovis* and *Mtb* H37Rv as stimulating antigens. γδ Tcell and NKT cell responses were evaluated using their cognate antigens (E)-4-hydroxy-3-methyl-but-2-enyl pyrophosphate (HMBPP) and α-galactosylceramide (α-GalCer), respectively. And stimulation with TB10.4 and *M.bovis* culture filtrate proteins (CFP) served as stimulants for assessing conventional peptide antigen-specific immune responses. IFN-γ release in response to ex vivo stimulation of PBMC with TB10.4 peptide pool was detectable by 3 weeks following BCG exposure (**Figure 5A**) and it declined by 7 weeks. CFP responses displayed more variability between individuals and followed a similar kinetics of increase around 3 weeks and decline by 7 weeks of BCG exposure (**Figure 5A**). Notably, a significant increase in IFN-γ release in response to *M.bovis* TL was observed earlier at 2 weeks post-BCG inoculation, although it returned to baseline by 7 weeks similar to TB10.4 and CFP responses (**Figure 5A**). IFN-γ response to Mtb TL, however, peaked at 3 weeks post-BCG in contrast to *M.bovis* TL (**Figure 5A**), suggesting qualitative differences in lipid-reactive T cell responses to closely related mycobacterial strains. This was consistent with peak IFN-γ response of PBMC to individual Mtb lipid fractions including LAM, Phosphoinositol mannoside-6 (PIM-6), and mycolic acids (MA) at 3 weeks post-BCG (**Supplementary Figure 2**). Interestingly, IFN-γ release by peripheral blood γδ T cells increased earlier than even *M.bovis* TL at 1-week post-BCG and were at a larger scale (mean = 3010 SFC/million PBMC at 1-week and 9858 SFC/million PBMC at 2-weeks post-BCG) compared to the other mycobacterial peptide and lipid antigens tested (950-1500 SFC/million PBMC at peak 3-week time-point) suggesting that γδ T cell responses form a dominant part of early immune response to BCG exposure. The kinetics of NKT cell IFN-γ responses, however, were similar to peptide-specific responses (**Figure 5A**). Notably, in BAL fluid, no responses were observed at baseline for both peptide and lipid-reactive cells besides innate-like γδ T cells and NKT cells responding to their cognate antigens HMBPP and α-GalCer (**Figure 5B**), suggesting that IFN-γ responses to mycobacterial antigens were more regulated in the airway than in peripheral blood. Although we anticipated earlier BAL responses to mycobacterial lipids owing to the aerosol exposure route, IFN-γ responses were more delayed in BAL starting around 3 weeks of BCG exposure. Further, these responses persisted longer for up to 7 weeks in BAL than blood despite clearance of live bacteria by the week-3 time-point (**Figure 5B**).

### Differential kinetics of IFN-γ response to peptide and lipid antigens in the airway following BCG exposure

**Figure 6:**
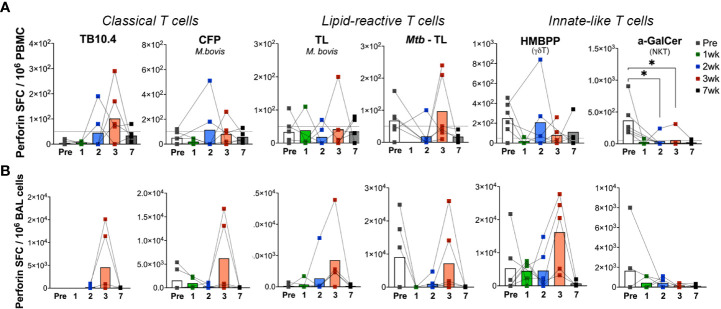
Kinetics of peptide- and lipid-reactive Perforin responses in blood and BAL following aerosol BCG exposure. Perforin production expressed as SFU/million cells, following stimulation with TB10.4 peptide pool, M.bovis CFP, M.bovis and Mtb TL fractions, HMBPP, and α-GalCer in freshly isolated **(A)** PBMCs, and **(B)** BAL lymphocytes. Pre-BCG inoculation (white bars) and 1wk (green bars), 2wk (blue bars), 3wk post-BCG (red bars), and 7wk (grey bars) time-points were evaluated. Wilcoxon matched pairs signed rank test was used to compute difference between time points with respect to pre-BCG baseline. Asterisks indicate significant differences between time points (*p < 0.05).

In order to understand the kinetics of unknown lipid-reactive T cell responses relative to well characterized peptide-specific responses, we had simultaneously included both peptide and lipid antigens in our ELISPOT assays. Besides the differing kinetics of lipid-reactive IFN-γ responses between blood and BAL, a significant difference was observed in the timing of peak responses in BAL to mycobacterial peptides in TB10.4 versus total lipids in our study. IFN-γ release following ex vivo *M.bovis* TL and *Mtb* TL stimulation was significantly higher at week-7 in BAL in contrast to TB10.4 responses peaking at week-3, suggesting the induction of longer-lasting Th1 type responses in local lipid-reactive T cells induced by BCG exposure in the airway. However, use of individual lipid antigens including *Mtb* LAM, PIM-6, and MA showed peak IFN-γ responses at week-3 (**Supplementary Figure 2**) that had reduced by week-7. IFN-γ responses of BAL γδ Tand NKT cells did not change significantly between pre- and post-BCG time-points (**Figure 5B**), although there was a trend for greater responses than peripheral blood at the 7-week time-point in concordance with the sustained IFN-γ responses observed in T cells responding to total mycobacterial lipids. The cytotoxic potential was further evaluated *via* Perforin ELISPOT. Perforin release in response to the peptide and lipid antigens followed a similar pattern to IFN-γ responses but displayed significant variability between individuals (**Figure 6**). Unlike IFN-γ responses, both Blood and BAL displayed maximal Perforin producing ability at week-3, subsequently declining to baseline by week-7 post-BCG inoculation (**Figures 6A, B**). Notably, BAL γδ T cells showed a trend of increase in Perforin production at week-3 post-BCG (**Figure 6B**). In contrast, there was a significant decline in perforin production by peripheral blood NKT cells (**Figure 6A**) and a similar yet insignificant trend in BAL fluid (**Figure 6B**).

### Systemic loss of IL-7R expression in γδ T cells along with increased GrB expression in BAL during SIV infection

**Figure 7:**
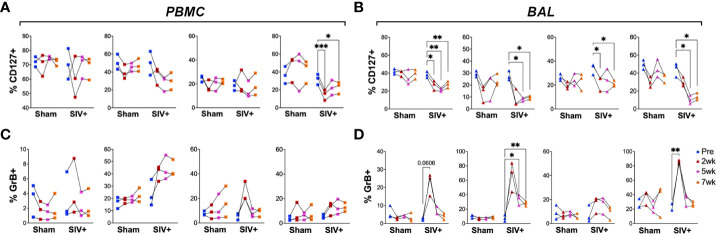
Impact of SIV infection on the expression of CD127 (IL-7R) and GrB on T cell subsets. Frequencies of CD4 T, CD8 T, DNT, and γδ T cells in PBMCs (left panels **A**, **C**) and BAL cells (right panels **B**, **D**) expressing CD127 (top) and GrB (bottom). Comparisons of different time points with respect to pre-SIV baseline was carried out using repeated measures one-way ANOVA with Dunnett’s *post hoc* tests. Asterisks indicate significant differences between time points (*p < 0.05; **p < 0.01; ***p < 0.001).

Human immunodeficiency virus (HIV) and simian immunodeficiency virus (SIV) coinfection have been shown to induce reactivation of latent TB *via* impairment of protective T cell functions and loss of immune control of the bacilli (6, 37–39). Peptide antigen-specific CD8T cells have been associated with loss of IL-7R and development of memory phenotype that correlate with markers of disease progression (i.e., plasma viremia and CD4T cell depletion) as well as with the overall levels of immune activation during HIV infection (40). To examine whether SIV infection also impacts lipid-reactive T cell functions, the BCG-inoculated macaques were divided into two groups, with one half receiving SIVmc251 challenge infection and the other half receiving sham treatment. SIV inoculation resulted in peak viremia (6.5–7.5 log SIV RNA copies/ml) between weeks 1-2 in all 3 macaques and dropped to set point viremia (4.4–6 log SIV RNA copies/ml) by 8 weeks post-SIV (**Supplementary Figure 3**). The presence of live mycobacteria was evaluated in BAL fluid at 3-, 5- and 8-weeks post-SIV time-points. No BCG was recovered from BAL at any of these time-points post-BCG, indicating that BCG was likely cleared in these animals prior to SIV infection. Longitudinal changes in the expression of HLA-DR, CXCR3 and CD127 were evaluated as earlier described in the BCG-inoculation phase. Additionally, intracellular expression of GrB was evaluated as a measure of cytotoxic functionality. NKT cells were not included owing to significant depletion by SIV as earlier established by us and others (41–43) resulting in very rare events confounding immunophenotyping analyses. Expression of CD127 was highly significantly reduced in peripheral blood γδ T cells by 2 weeks of SIV infection (P=0.0003) and failed to recover to baseline levels during chronic SIV infection as seen at the week-7 timepoint (**Figure 7A**). In BAL fluid, however, the persistent loss in CD127 expression was universal on all T cell subsets (**Figure 7B**). On the other hand, GrB expression increased transiently, yet significantly in BAL γδ T cells early at 2 weeks post-SIV (P=0.008) and was more persistent in CD8T cells between 5-7 weeks post-SIV infection (**Figure 7D**), suggesting SIV-induced induction of effector functions in γδ T cells and CD8T cells in airway.

### Impairment of IFN-γ responses of lipid-reactive T cells in BAL fluid parallels the loss of peptide-specific responses during SIV infection

**Figure 8:**
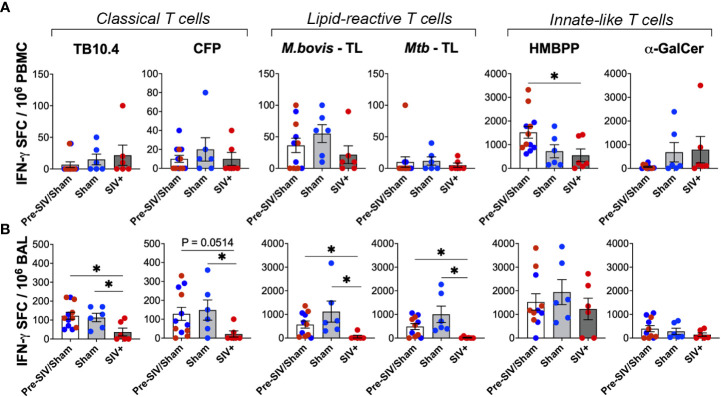
Loss of peptide- and lipid-reactive IFN-γ responses in BAL fluid of macaques following SIV infection. Quantification of IFN-γ producing cells expressed as SFU/million cells, following stimulation with TB10.4 peptide pool, *M.bovis* CFP, *M.bovis* and *Mtb* TL fractions, HMBPP, and α-GalCer in freshly isolated **(A)** PBMCs, and **(B)** BAL lymphocytes. 1wk pre-SIV/sham inoculation (white bars), and 6-8 weeks post SIV/sham (grey bars) show individual macaques inoculated with either sham (blue circles) or SIV (red circles). Unpaired t test was used to compute differences between groups and with respect to pre-SIV/sham baseline. Asterisks indicate significant differences between time points (*p < 0.05).

To further assess the impact of SIV infection on the IFN-γ secreting ability of lipid-reactive T cells, we evaluated them along with classical TB10.4 and CFP responses using IFN-γ-ELISPOT assay. Due to lower cell yields and the unavailability of sufficient cells for every animal at a given time-point, combined time-points pre-SIV (within one week of SIV challenge) and post-SIV (6-8 weeks post-SIV) were compared cross-sectionally between the pre-SIV, post-SIV and sham treatment groups. To avoid duplication, no animal was repeated for a single time-point. As anticipated, pre-SIV/sham treatment IFN-γ SFC counts were normally distributed between the animals (**Figures 8A, B**). There was no notable change in IFN-γ SFC counts in PBMCs stimulated with either TB10.4 and CFP, or mycobacterial total lipids (**Figure 8A**). Only IFN-γ response of γδ T cells to HMBPP stimulation was significantly lower in PBMCs of SIV-infected macaques but maintained in BAL (**Figures 8A, B**). Notably, in BAL fluid, chronic SIV infection (6-8 weeks post-SIV) induced a significant reduction in numbers of IFN-γ secreting cells responding to total lipids of *M. bovis* and *Mtb*, as well as the peptide antigens TB10.4 and CFP (**Figure 8B**). No significant changes from pre-SIV/sham values were observed in the time-matched sham group that received saline injections. This suggested that in the airway, SIV infection induced a more pronounced impairment in IFN-γ secreting functions of mycobacterial antigen-specific T cells than innate-like T cells that display a broader recognition.

### Maintained cytotoxic functions of mycobacterial lipid-reactive T cells during SIV infection

**Figure 9:**
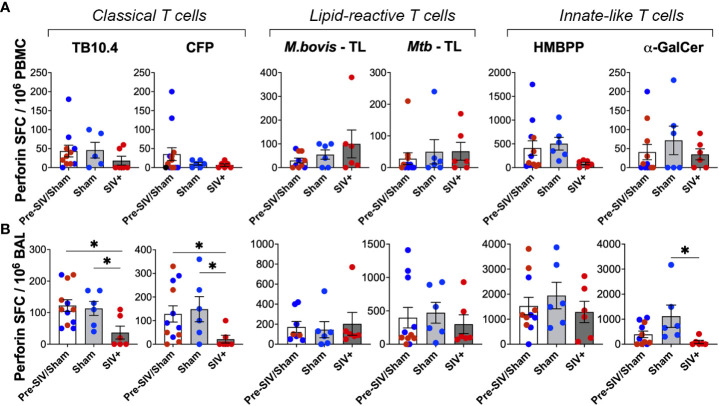
No impact of chronic SIV infection on Perforin production by lipid-reactive T cells in BAL fluid. Quantification of Perforin producing cells expressed as SFU/million cells, following stimulation with TB10.4 peptide pool, *M.bovis* CFP, *M.bovis* and *Mtb* TL fractions, HMBPP, and α-GalCer in freshly isolated **(A)** PBMCs, and **(B)** BAL lymphocytes. 1wk pre-SIV/sham inoculation (white bars), and 6-8 weeks post SIV/sham (grey bars) show individual macaques inoculated with either sham (blue circles) or SIV (red circles). Unpaired t test was used to compute differences between groups with respect to pre-SIV/sham baseline. Asterisks indicate significant differences between time points (*p < 0.05).

We next assessed the Perforin secreting ability of lipid-reactive T cells and compared them with classical TB10.4 and CFP responses between the SIV-infected and sham groups. Perforin SFC counts in PBMCs did not differ significantly following SIV infection for both peptide- and lipid-antigen responses, although there was a trend of decrease in TB10.4 and CFP responses (**Figure 9A**). However, classical T cell responses to TB10.4 and CFP stimulation in BAL fluid were significantly lower in chronic SIV infected animals in comparison to pre-SIV/sham responses (**Figure 9B**). A significantly lower Perforin response in BAL fluid of SIV-infected macaques was also noted for α-GalCer stimulation (**Figure 9B**) in concordance with the established loss of NKT cells during SIV infection (42, 44). Notably, unlike the classical TB10.4 and CFP responses, Perforin response to *M. bovis* and *Mtb* TL stimulation of BAL fluid was not impacted following SIV infection (**Figure 9B**) suggesting the maintenance of cytotoxic functionality in mycobacterial lipid-reactive T cells during chronic SIV infection. Perforin response of γδ T cells to HMBPP stimulation was also maintained in BAL fluid of SIV-infected macaques at levels similar to that in sham group, although there was a trend of decline in PBMCs (**Figures 8A, B**). Taken together with increased *in vivo* GrB expression by BAL γδ T cells at 2 weeks post-SIV, these functional data indicate that although there is a significant loss of Th1-type IFN-γ effector functions in overall antigen-specific T cells following SIV infection, the cytotoxic effector functions mycobacterial lipid-reactive T cells are maintained at least during post-acute peak viremia in BCG-inoculated macaques.

## Discussion

Seminal studies focused on unconventional T cells in Mtb-infected persons including HIV-infected individuals have provided important cues into the function of this arm of immunity in TB (23, 45–48). However, it is challenging to address their *in vivo* response to Mtb exposure in humans, particularly in the lungs during early stages of infection due to the inability to predict new infections in a non-controlled setting. Our study, broadly focused on mycobacterial lipid-reactive T cell responses in macaques as the nonhuman primate model of TB/HIV infection, demonstrates the presence of innate-like mycobacterial lipid-reactive T cells that display distinct kinetics of response from that of conventional peptide-specific T cell responses to live mycobacterial exposure in the airways. Further, our results suggest that lipid-reactive T cells may contribute to systemic anti-mycobacterial cytolytic functions during chronic SIV infection. Thus, the induction of durable cross-reactive mycobacterial lipid-specific T cell functions could have potential implications on the initial immune response to pathogenic mycobacterial exposure.

We selected the aerosol BCG inoculation route to model controlled pulmonary mycobacterial infection in macaques, since it represents the natural route of Mtb infection and aerosol delivery of BCG has been shown to be safe, immunogenic and protective in rhesus macaques (49, 50). An interesting finding in our studies is the detection of mycobacterial lipid-reactive IFN-γ and GrB-producing T cells in peripheral blood of BCG-naïve, TST-negative macaques. This is perhaps not surprising given that mycobacterial glycolipids present in the cell wall of several non-pathogenic species including *M. smegmatis* share epitopes with Mtb and BCG (51). Although the lipid-rich mycobacterial cell wall components initially interact with classical antigen presenting cells, particularly macrophages and dendritic cells (DC) (52–54) that consequently trigger the T cell-mediated immune response, we believe that the ex vivo responses of PBMCs and BAL cells in our study were mainly contributed by lipid-reactive T cells based on our intracellular cytokine staining results demonstrating recent activation and increase in IFN-γ and GrB production in T cells following lipid stimulation. Moreover, owing to lower frequencies, macrophages and DCs typically need *in vitro* expansion prior to stimulation with mycobacterial lipid antigens for the evaluation of detectable cytokine/cytolytic secretion (53–55). Thus, the presence of circulating mycobacterial lipid-reactive IFN-γ and GrB T cells in BCG-naïve macaques is indicative of previous exposure to nontuberculous mycobacteria in the environment and likely represents cross-reactive T cells similar to the reported purified protein derivative (PPD)-induced IFN-γ responses in tuberculin skin test (TST)-negative healthy persons without Mtb infection or BCG vaccination (56). Further, our results are in concordance with the previously reported IFN-γ-producing and cytolytic T cell responses to mycobacterial lipids, particularly LAM, in humans as a part of the CD1-restricted T cell recognition of microbial antigens (24). Interestingly, the dominant proportions of CD8-positive and CD4/CD8-double negative T cell subsets in the mycobacterial lipid-reactive T cells suggest that majority of these cells are CD4-negative and thus non-targets for direct HIV/SIV infection. This is critical in the setting of HIV coinfection since HIV-induced increase in TB risk is mainly attributed to loss of CD4 T cell functions (57, 58) and the residual anti-mycobacterial T cell responses are dependent on non-CD4 T cell effector functions. Given the presence of human CD4 T cell responses to the mycobacterial cell wall lipids including mycolic acid (MA) and glucose monomycolate (GMM) (48, 59, 60), our results suggest that the dominant lipid antigens presented in total lipid fractions of *M. bovis* and Mtb are recognized by CD8 T cells and DNT cells.

γδT cells form a major part of unconventional lipid-reactive T cells in the human cellular immune system and recognize lipid antigens presented on the nonpolymorphic MHC class I–like CD1 molecules (61–64). Accordingly, *in vivo* expansion of γδ T cells have been recently reported in the blood and lungs of nonhuman primates after intravenous administration of BCG (59, 65, 66). However, previous studies have explored γδ T cells and other CD1-restricted T cells starting from 2-3 weeks till up to 1-year post-inoculation (65, 67, 68). Our study is the first to assess very early T cell responses (between days 1-7) to BCG exposure in macaques and demonstrates a significant increase in γδ T cells in the airways within a day of aerosol BCG exposure, which supports their innate-like responses to mycobacterial antigens. However, it is not clear whether this was the result of local expansion or recruitment of peripheral γδ T cells since there was no notable change in circulating γδ T cell frequencies following aerosol BCG exposure. The concomitant increase in IL-7R and subsequent CXCR3 expression of BAL γδ T cells post-BCG inoculation support their activation and induction of Th1-type central memory phenotype in the airway mucosa. Since IL-7R signaling is essential for the generation of memory T cells (69, 70), its rapid induction on airway γδ T and NKT cells, two well-characterized subsets of CD1-restricted T cells, suggests that lipid-reactive T cells may be involved in the initial recall responses to subsequent mycobacterial infections. Indeed, lipid-reactive (mycolic acid-specific) T cells in TB patients were shown to exhibit recall responses upon *in vitro* antigen restimulation long after successful treatment and contraction of the initial responses during active TB, showing presence of lipid-specific immunological memory (68). T cell memory in the lungs is critical in protection against Mtb and vaccination with BCG supplemented with IL-7 and IL-15 was shown to induce greater proliferation of T cells and the predominant release of Th1-like cytokines in the lungs (70). Thus, based on the early induction of IL-7R and CXCR3 expression on lipid-reactive T cells in our study, we speculate that specific mycobacterial lipids may be involved in the acute and memory immune response to BCG vaccination in humans.

In the setting of cleared BCG infection in nonhuman primates, our study demonstrated longer persistence of mycobacterial lipid-induced IFN-γ responses targeted toward BCG and Mtb TL in the BAL fluid than in blood. Further, the earlier detection of ex vivo IFN-γ responses to *M. bovis* TL at week-2 in comparison to Mtb TL responses at week-3 post-BCG inoculation suggest that there may be strain-specific differences in the lipids presented (71) and that broader cross-reactive responses likely develop later during mycobacterial infection. Previous studies have reported the generation of cross-reactive responses against Mtb antigens by the cell wall components of non-pathogenic mycobacteria including *M. smegmatis* and BCG and demonstrated that immunogenic glycolipids in *M. smegmatis* were capable of inducing antibody response to surface antigens of Mtb (51, 72). Further, it is interesting to note that the cytolytic functions of lipid-reactive T cells induced by aerosol BCG exposure were less durable than IFN-γ responses in the lungs and waned similar to TB10.4 and CFP responses, suggesting that their cytolytic functions may require presence of live bacteria given that BCG was cleared from the study animals by 3 weeks of inoculation.

Although our study was limited by numbers of three animals per group following SIV infection, the differences between the groups are consistent for phenotypic and functional markers suggesting partial impairment of lipid-reactive T cells induced by SIV infection. The acute loss of IL-7R expression on blood and BAL γδ T cells along with a broader decline in all T cell subsets in BAL fluid following SIV infection in our study is consistent with the established impact of HIV and SIV infections on T cells (40, 73). The impact of SIV infection was also observed in IFN-γ responses of BAL lymphocytes to both peptide antigens in TB10.4 as well as total lipids, which likely contributes to the loss of immune control of TB in the setting of HIV/SIV-induced reactivation of latent TB. The maintained cytolytic ability of lipid-reactive T cells *via* Perforin secretion during SIV infection, however, is interesting since cytolytic effector functions toward mycobacterial cell wall lipids likely play a crucial role in the initial response to Mtb infection or latent TB reactivation in the lungs. Cytolytic functions have been shown to be an important component of TB control in several studies (74–76). Future studies with low-dose Mtb to establish latent TB followed by SIV challenge, as earlier described (77), are required to understand the role of lipid-reactive T cell functions in the breakdown of control of TB infection.

In summary, our study demonstrated that mycobacterial lipid-reactive T cells display early innate-like effector functions in peripheral blood of BCG-naïve macaques and these functions are enhanced by aerosol BCG exposure. While SIV infection disrupted cytokine function and memory generation in mycobacterial lipid-reactive T cells, their cytolytic potential was maintained, suggesting that targeting this function by specific mycobacterial lipids as adjuvants may be incorporated into future vaccine design.

## Data availability statement

The original contributions presented in the study are included in the article/**Supplementary Material**. Further inquiries can be directed to the corresponding author.

## Ethics statement

The animal study was reviewed and approved by Tulane University Institutional Animal Care and Use committee (IACUC).

## Author contributions

NR conceived the project, designed experiments, acquired funding, and supervised the work. EW, KM, NS performed experiments and analyzed data. BG performed the animal experiments in this study. DK and SM supplied BCG and provided technical guidance for bacterial burden determination. CR supervised the aerosol delivery of BCG into macaques. DK provided conceptual insights. All authors read and approved the final manuscript.
