# Supplementary material for: Impact of SIV infection on mycobacterial lipid-reactive T cell responses in Bacillus Calmette-Guérin (BCG) inoculated macaques
Source: Front Immunol. 2023 Jan 16;13:1085786. doi: 10.3389/fimmu.2022.1085786 (PMC9885173; doi:10.3389/fimmu.2022.1085786)
Supplement: Supplementary file 1 [file DataSheet_1.docx]

Supplementary Material


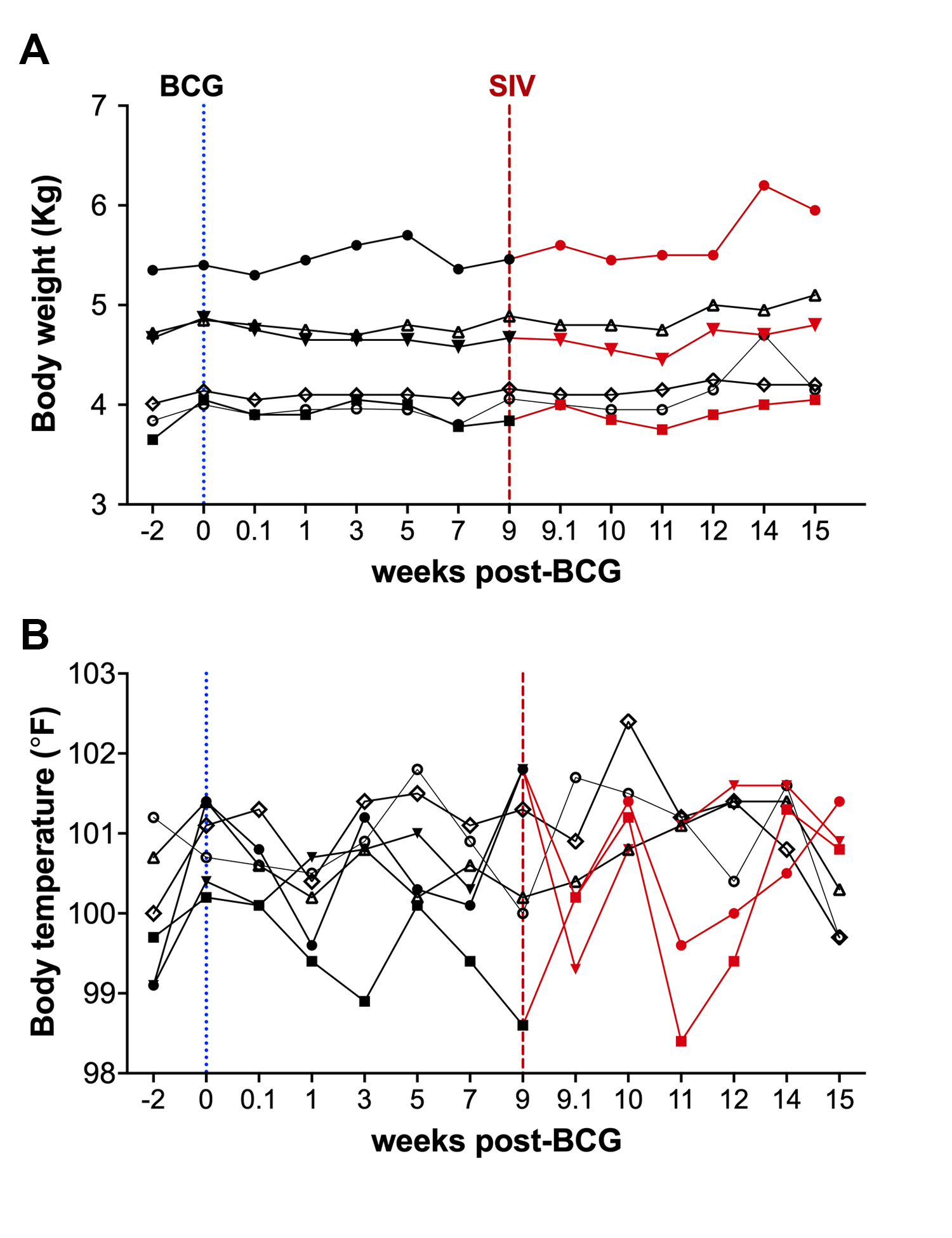


**Supplementary Figure 1. Changes in weight and temperature during BCG/SIV infection in macaques.** Longitudinal assessment of (A) Weight and (B) Temperature in cynomolgus macaques measured pre- and post-BCG inoculation and until week-15. Three animals given intravenous challenge SIV infection at week 9 post-BCG are shown in red. The remaining animals received saline injections at week 9. No significant changes were observed in body weight or temperature following infection.


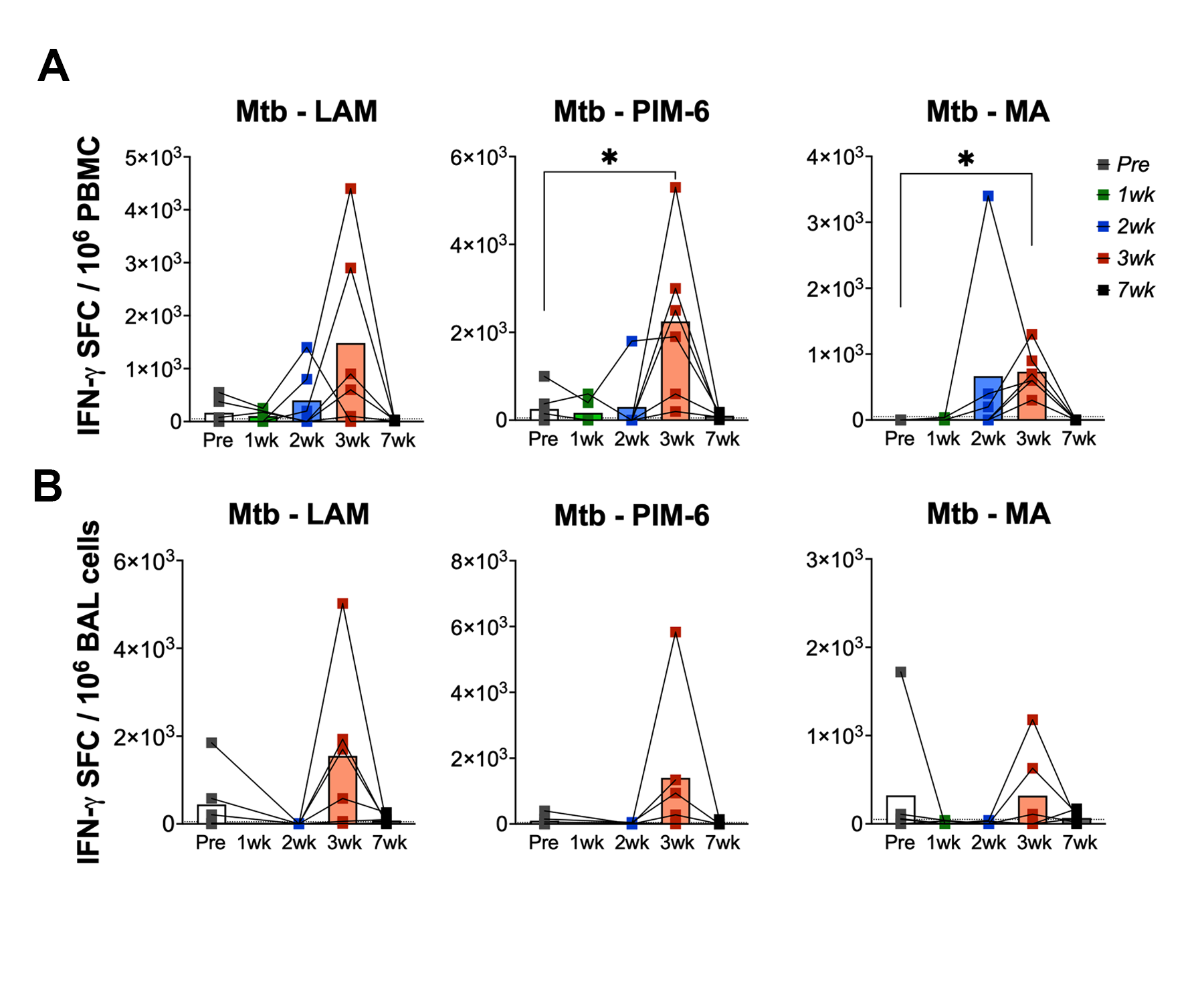


**Supplementary Figure 2. Kinetics of Mtb lipid-reactive IFN-γ responses in blood and BAL following aerosol BCG exposure.** IFN-γ responses expressed as SFU/million cells, following stimulation with *Mtb* lipids: Lipoarabinomannan (LAM), Phosphoinositol mannoside-6 (PIM-6), and mycolic acids (MA), in freshly isolated (A) PBMCs, and (B) BAL lymphocytes. Pre-BCG inoculation (white bars) and 1wk (green bars), 2wk (blue bars), 3wk post-BCG (red bars), and 7wk (grey bars) time-points were evaluated. Wilcoxon matched pairs signed rank test was used to compute difference between time points with respect to pre-BCG baseline. Asterisks indicate significant differences between time points (*p < 0.05).


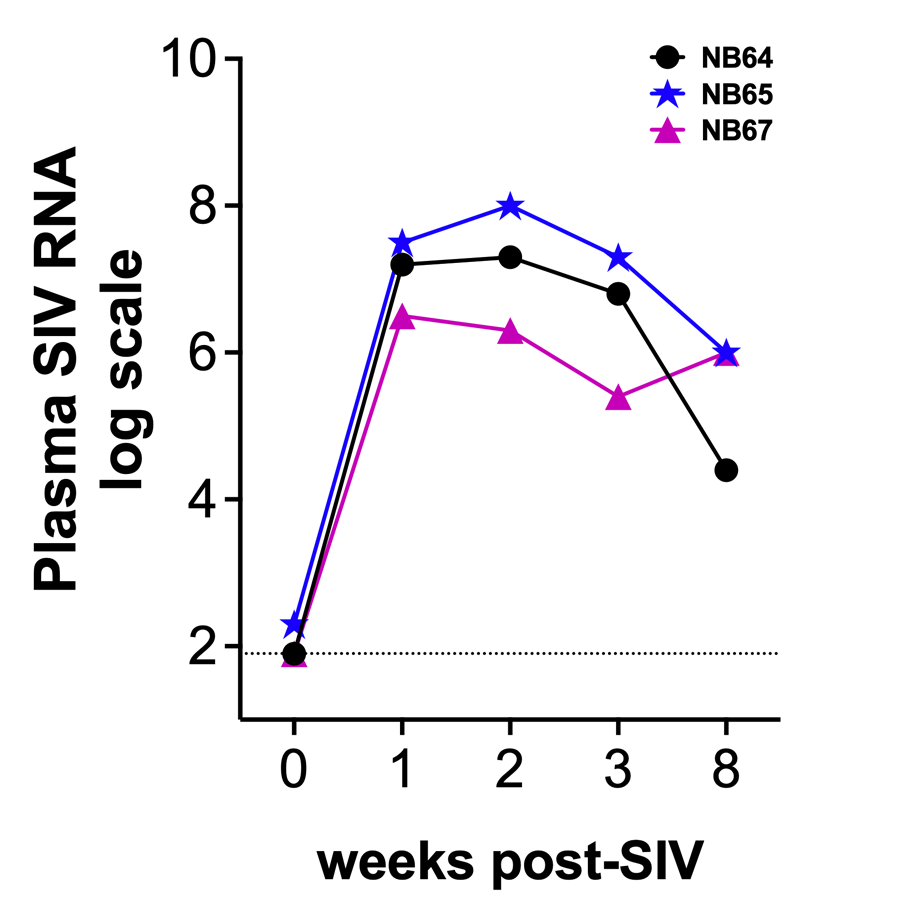


**Supplementary Figure 3. Plasma viral loads.** Longitudinal SIV RNA quantification in plasma showing peak viremia at 1-2 weeks post-SIV infection and decline to set-point viremia by 8 weeks of infection.
